# Maturation and Conformational Switching of a *DeNovo* Designed Phase-Separating Polypeptide

**DOI:** 10.1021/jacs.4c00256

**Published:** 2024-04-05

**Authors:** Alexander T. Hilditch, Andrey Romanyuk, Lorna R. Hodgson, Judith Mantell, Christopher R. Neal, Paul Verkade, Richard Obexer, Louise C. Serpell, Jennifer J. McManus, Derek N. Woolfson

**Affiliations:** † School of Chemistry, University of Bristol, Cantock’s Close, Bristol BS8 1TS, U.K.; ‡ Max Planck-Bristol Centre for Minimal Biology, University of Bristol, Cantock’s Close, Bristol BS8 1TS, U.K.; § Wolfson Bioimaging Facility, University of Bristol, Biomedical Sciences Building, Bristol BS8 1TD, U.K.; ∥ School of Biochemistry, University of Bristol, Biomedical Sciences Building, Bristol BS8 1TD, U.K.; ⊥ Bristol BioDesign Institute, School of Chemistry, University of Bristol, Cantock’s Close, Bristol BS8 1TS, U.K.; # Department of Chemistry, Manchester Institute of Biotechnology, University of Manchester, Princess Street, Manchester M1 7DN, U.K.; ⊗ School of Life Sciences, University of Sussex, Falmer, Brighton, JMS 3B17, U.K.; ¶ HH Wills Physics Laboratory, School of Physics, University of Bristol, Tyndall Avenue, Bristol BS8 1TL, U.K.

## Abstract

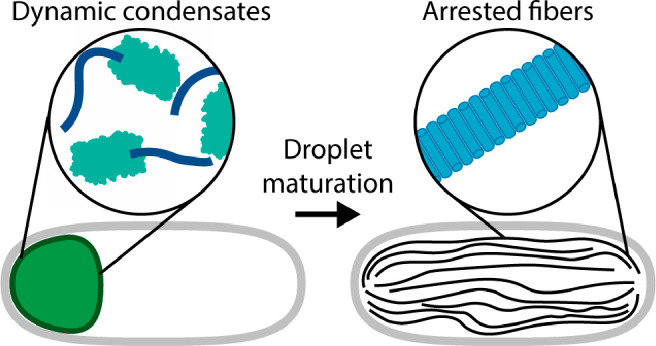

Cellular compartments formed by biomolecular condensation are widespread features of cell biology. These organelle-like assemblies compartmentalize macromolecules dynamically within the crowded intracellular environment. However, the intermolecular interactions that produce condensed droplets may also create arrested states and potentially pathological assemblies such as fibers, aggregates, and gels through droplet maturation. Protein liquid–liquid phase separation is a metastable process, so maturation may be an intrinsic property of phase-separating proteins, where nucleation of different phases or states arises in supersaturated condensates. Here, we describe the formation of both phase-separated droplets and proteinaceous fibers driven by a *de novo* designed polypeptide. We characterize the formation of supramolecular fibers *in vitro* and in bacterial cells. We show that client proteins can be targeted to the fibers in cells using a droplet-forming construct. Finally, we explore the interplay between phase separation and fiber formation of the *de novo* polypeptide, showing that the droplets mature with a post-translational switch to largely β conformations, analogous to models of pathological phase separation.

Membraneless organelles (MLOs) formed by biomolecular condensation are now recognized as a widespread phenomenon in cell biology.^1^ Macromolecular condensation is implicated in many cellular processes, from chromatin organization, cell signaling, and genetic regulation, to microtubule organization, cell division, and cellular stress responses.^2−5^ Nonetheless, there are still significant gaps in our knowledge of how phase-separating proteins are organized and controlled at a molecular level.^6^ Recent advances in understanding the molecular driving forces of protein phase separation have come from the bottom-up design of artificial biomolecular condensates.^7^ These offer the potential to create synthetic MLOs, orthogonal to endogenous cellular processes. While phase separation is being explored for its potential to augment biology, the same processes are being interrogated clinically for their roles in disease and degenerative processes, such as frontotemporal dementia (FTD), and amyotrophic lateral sclerosis (ALS).^8^

In protein phase separation, weak attractive intermolecular interactions produce demixed droplets in which macromolecules are enriched, but still diffuse quickly.^9^ However, these same interactions can also lead to arrested proteinaceous assemblies, which can be nucleated from phase-separated droplets.^10^ This secondary formation of a new protein state has been termed the maturation or molecular aging of protein condensates.^11^ In some cases, droplet maturation is an essential part of biological function.^12^ However, droplet maturation has been implicated in the formation of pathological proteinaceous assemblies, including amyloid fibers and aggregates.^13,14^ Moreover, several ALS-related mutations in phase-separating proteins have been observed to accelerate the transition from dynamic to arrested states.^10,15,16^ Yet, the mechanisms that lead to droplet maturation are relatively poorly understood. Some maturation events have been linked to specific sequence elements, or changes in the cellular environment.^17^

Understanding how droplet maturation occurs could lead to more effective treatment of amyloid pathologies.^18^ Previously, we reported a *de novo* polypeptide, HERD-2.2, for liquid–liquid phase separation (LLPS, Table S1).^19^ Here, we describe the interplay between the phase separation and fiber formation of this synthetic polypeptide. HERD-2.2 was designed to function as a genetic fusion to client proteins such as fluorescent reporters or enzymes. For instance, a fusion to the green fluorescent protein (GFP) mEmerald, denoted HERD-2.2–GFP, phase separates as demixed droplets *in vitro* and in *Escherichia coli* (*E. coli*) (Figures 1A and S1). Here we describe the phase behavior of the HERD-2.2 module in isolation.

**Figure 1:**
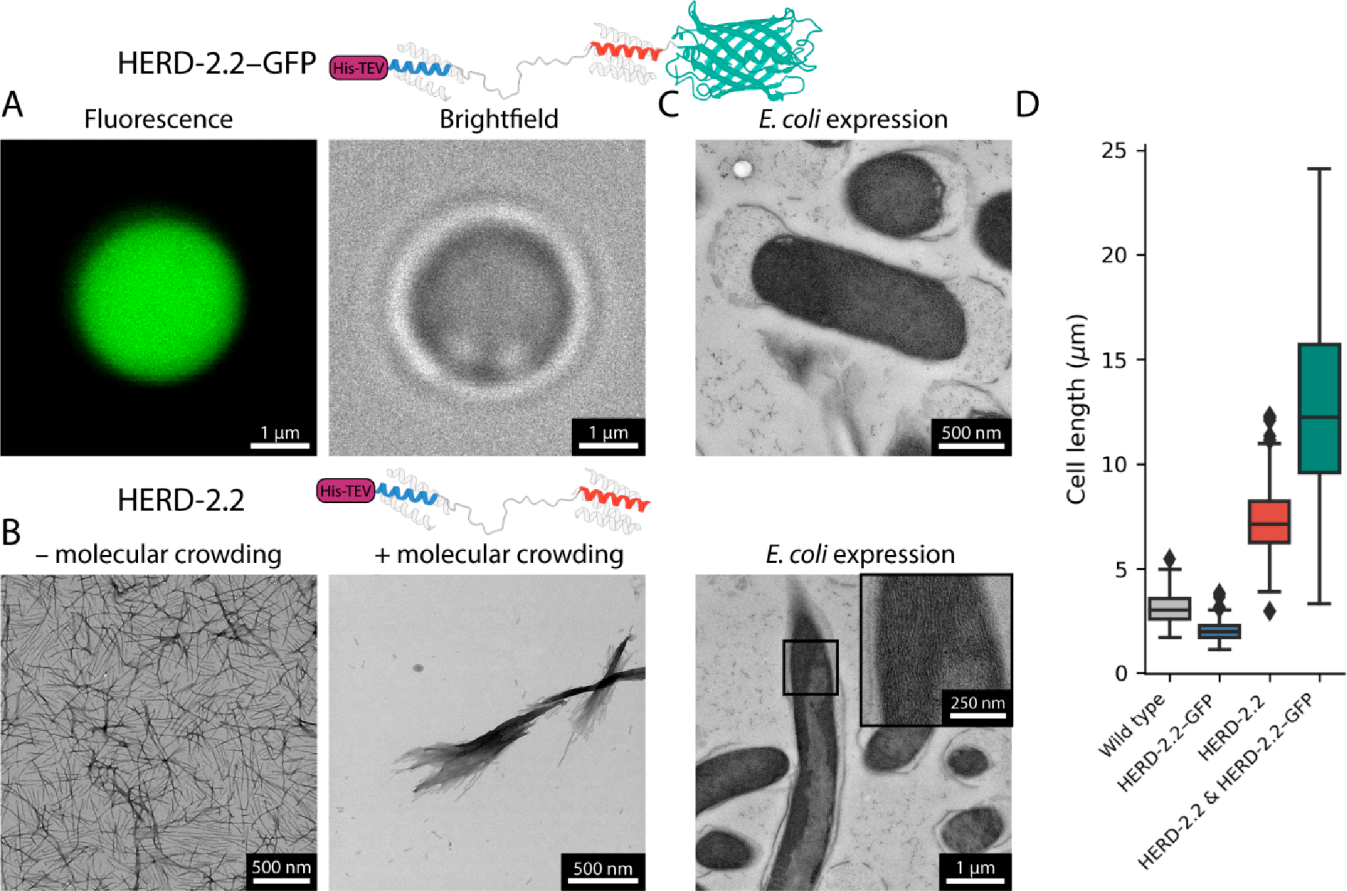
HERD-2.2 forms supramolecular fibers *in vitro* and in *E. coli*. (A) Confocal microscopy images of HERD-2.2–GFP. Conditions: 110 μM HERD-2.2–GFP, 10% PEG 3350, 125 mM NaCl, 50 mM Tris pH 7.5. (B) Negative stain TEM images of HERD-2.2 without molecular crowding (left; 25 μM HERD-2.2, 50 mM Tris pH 7.5) and with (right; 25 μM HERD-2.2, 10% PEG 3350, 125 mM NaCl, 50 mM Tris pH 7.5). (C) TEM sections of *E. coli* expressing HERD-2.2–GFP and HERD-2.2. (D) Cell length of WT *E. coli* (*n* = 106), *E. coli* expressing HERD-2.2–GFP (*n* = 100), HERD-2.2 (*n* = 107), and HERD-2.2–GFP and HERD-2.2 coexpressed (*n* = 100).

The isolated HERD-2.2 polypeptide was expressed and purified from *E. coli* using an *N*-terminal His tag, and phase separation was probed *in vitro*. Instead of forming demixed droplets, HERD-2.2 formed supramolecular fibers (Figures 1B and S2). The HERD-2.2 fibers were examined by negative stain transmission electron microscopy (TEM). This revealed dispersed fibers in dilute solution with a mean diameter of 13 nm (Figure S3). However, with the addition of molecular crowding agents to mimic the cytoplasmic environment and as used to phase separate HERD-2.2–GFP (10% PEG 3350, 125 mM NaCl, 50 mM Tris pH 7.5), the fibers assembled further and laterally to give structures several micrometers in length and of variable thickness (Figure S4).

Having observed HERD-2.2 fibers *in vitro*, we looked directly in cells. Sections of *E. coli* expressing HERD-2.2 were imaged by TEM, confirming fiber formation within the cytoplasm (Figures 1C and S5). These fibers assembled laterally, analogously to those seen *in vitro* with molecular crowding agents. Cells expressing HERD-2.2–GFP did not form fibers but instead the previously reported dense regions of protein at the cell poles (Figures S6 and S7). Moreover, cells expressing HERD-2.2 grew much longer than the expected length for *E. coli* with a mean length of 7.4 ± 1.8 μm (Figure 1D).

The formation of such large fibers within the *E. coli* cytoplasm is remarkable, and reminiscent of engineered systems for subcellular recruitment using fibrous assemblies.^20^ Therefore, we assessed the capacity for HERD-2.2 fibers to act as a recruitment scaffold but in this case for other assemblies. To this end, HERD-2.2 and HERD-2.2–GFP were coexpressed in *E. coli*. SDS-PAGE indicated that both proteins were expressed, with HERD-2.2 in excess of HERD-2.2–GFP (Figure S8). This coexpression produced fluorescently labeled fibers, demonstrating that GFP had been recruited to the scaffold fibers (Figure S9). These cells shared the same elongated phenotype as cells expressing HERD-2.2 alone, with a mean length of 12.8 ± 4.5 μm (Figure 1D). Moreover, nucleic acid staining indicated multiple nucleoid regions, suggesting that the nucleoid had been segmented by fiber formation, though it does not appear to have impeded culture growth or protein expression (Figures S10 and S11). To confirm recruitment of HERD-2.2–GFP to the intracellular fibers, cells coexpressing HERD-2.2 and HERD-2.2–GFP were examined by correlative light and electron microscopy (CLEM). This revealed fluorescent HERD-2.2–GFP enriched along the HERD-2.2 fibers in 93% of cells (*n* = 30, Figure 2A), while expression of HERD-2.2–GFP alone produced fluorescence only around the condensates at the cell poles (Figure S12). Moreover, HERD-2.2 coexpressed with both HERD-2.2–GFP and HERD-2.2–mCherry showed enrichment of both fluorescent proteins, while coexpression with nontagged GFP and HERD-2.2–mCherry only the latter was recruited to the fibers (Figure S13).

**Figure 2:**
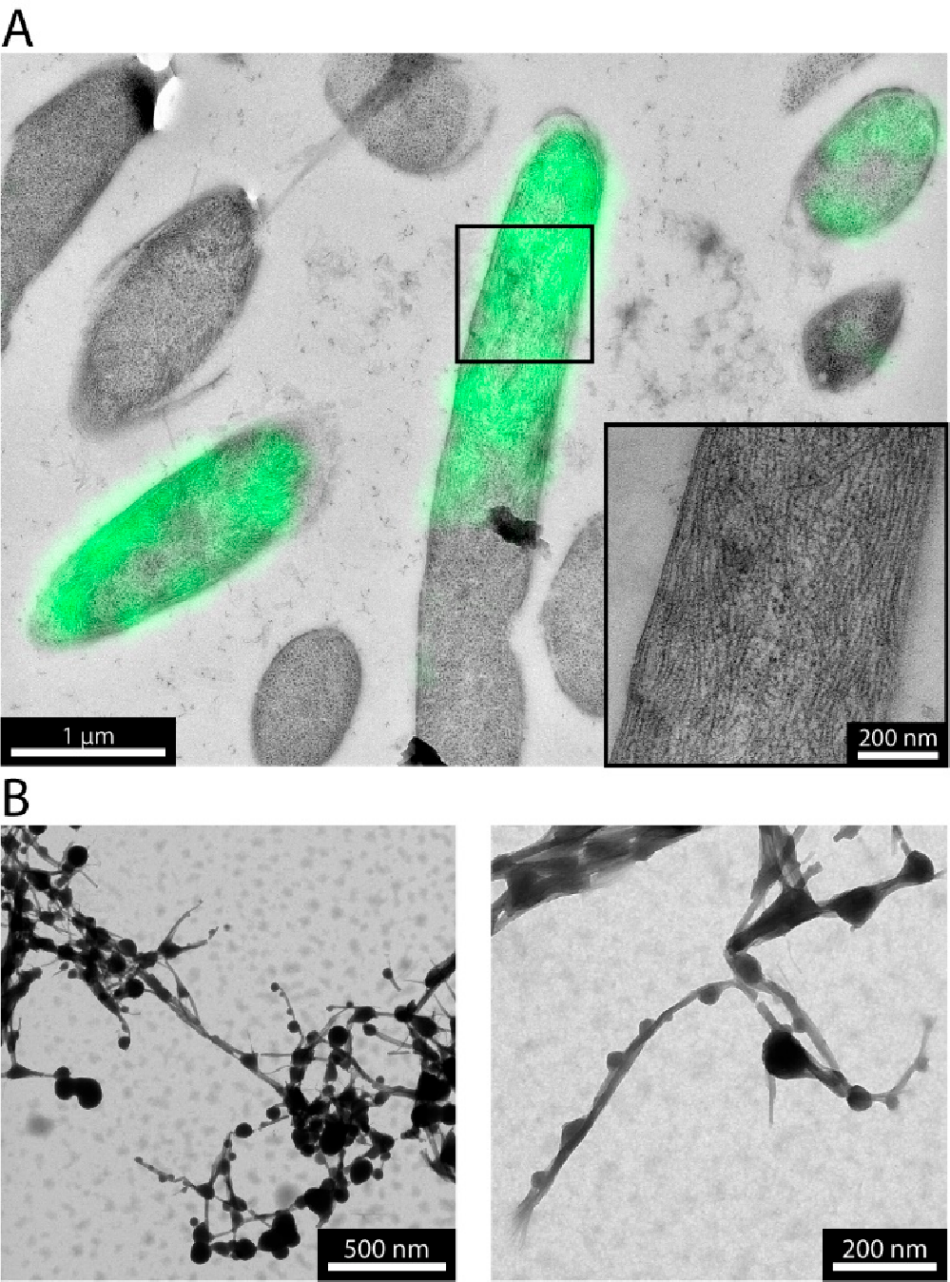
HERD-2.2 fibers labeled with droplet-forming HERD-2.2–GFP. (A) CLEM images of *E. coli* coexpressing HERD-2.2 and HERD-2.2–GFP. (B) TEM images of mixtures of HERD-2.2 and HERD-2.2–GFP. Conditions: 82 μM HERD-2.2, 55 μM HERD-2.2–GFP, 10% PEG 3350, 125 mM NaCl, 50 mM Tris pH 7.5.

Next, we examined the HERD-2.2–GFP and HERD-2.2 composites *in vitro*. Individually, these formed demixed liquid droplets and elongated fibers, respectively. When combined, and with molecular crowding regents, fluorescently labeled fibers were formed as observed in *E. coli*. However, these cell-free experiments revealed that the fluorescent protein was not distributed evenly but formed puncta along the fibers (Figure S14). This indicates that multiphase assemblies of both fibrous and droplet-like structures can be formed independently, but that they have an affinity for each other, which we suggest arises from the common HERD-2.2 component. TEM of these mixtures confirmed the formation of laterally assembled elongated fibers decorated with spherical droplet-like structures along their lengths (Figures 2B, S4, and S14).

Having observed fiber assembly, we interrogated their molecular structure. Initially, we postulated that the fibers could be α helical, as helical protein–protein interactions were used to design the HERD-2.2 fusions.^19^ Therefore, we examined the structure of the fibers by circular dichroism (CD) spectroscopy. CD spectra of the fibers in solution at 5 °C showed a weak shoulder around 220 nm (Figure 3A). Deconvolution of the spectrum indicated largely unstructured protein, with some α and β contributions (Figure S15). Heating the sample from 5 to 90 °C resulted in the irreversible loss of the shoulder and the α-helical contribution of the deconvolution (Figure S15), with a melting temperature of 58.0 ± 0.5 °C (Figure 3B). TEM before and after melting confirmed the loss of fibrous structures (Figure S16). However, cooling back to 5 °C did not induce refolding, nor the reformation of fibers, even after prolonged incubation (Figure S15). Addition of molecular crowding agents accentuated the shoulder around 220 nm, with a slight red-shift indicative of larger assemblies (Figure S17). To probe the fiber structure further, we used X-ray fiber diffraction (Figure S18). Diffraction of dried HERD-2.2 fibers gave a strong meridional reflection at 4.72 Å, characteristic of a cross-β structure (Figures 3C and S19).^21,22^ It is possible that this β structure was a consequence of partially drying the sample under tension. Therefore, we investigated the interaction of HERD-2.2 fibers with Thioflavin T (ThT), which shows increased fluorescence on interaction with amyloid-like fibrils.^23^ As a control, we also incubated ThT with a *de novo* dimeric α-helical coiled-coil peptide CC-Di.^24^ Addition of HERD-2.2 to 25 μM solutions of ThT resulted in a linear increase in ThT fluorescence proportional to the HERD-2.2 concentration, as seen for amyloid fibrils (Figure 3D).^25^

**Figure 3:**
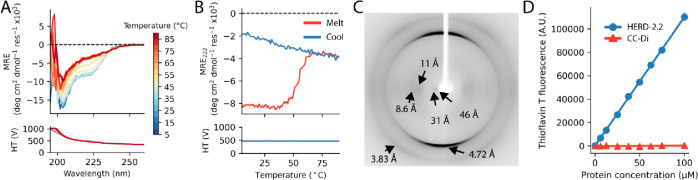
Biophysical characterization of the HERD-2.2 secondary structure. (A) CD spectra of HERD-2.2 on heating from 5–90 °C. Conditions: 25 μM HERD-2.2, 50 mM Tris pH 7.5. (B) CD of HERD-2.2 at 222 nm on heating and cooling. (C) X-ray fiber diffraction pattern of HERD-2.2. (D) Thioflavin T fluorescence with addition of HERD-2.2 or CC-Di.

Moreover, the fold-increase in ThT fluorescence closely matched that measured for the amyloid fibrils Aβ40 and Aβ42 (approximately 3.1-fold at 8 μM protein).^25^ In contrast, addition of CC-Di gave no increase in ThT fluorescence, even with up to 100 μM peptide.

This possible amyloid-like structure was surprising, as it is not fully consistent with CD spectra of the peptide constituents.^19^ Nonetheless, it illustrates that a structural transition to β is possible in these constructs. Moreover, the β secondary structure suggests that the HERD-2.2 fibers may mimic amyloid-like assemblies formed by natural phase-separating proteins;^26,27^ although the fibers formed by HERD-2.2 are more thermally labile than is usually observed (Figure 3B).^28,29^

The formation of both droplets and fibers by a designed protein could provide an accessible system for studying droplet maturation. To explore this further, we assessed whether HERD-2.2 could be switched from liquid-like droplets to fibers *in situ* by post-translational processing. The key difference between the two phase behaviors is the addition of the GFP *C*-terminal to the HERD-2.2 polypeptide. Therefore, to introduce a triggerable response, we inserted a thrombin cleavage site between HERD-2.2 and GFP, forming HERD-2.2-T–GFP. As with the parent HERD-2.2–GFP, addition of molecular crowding agents caused HERD-2.2-T–GFP to demix and form fluorescent droplets *in vitro* (Figure 4A). Next, we added the protease thrombin to cleave the *C*-terminal fluorescent protein. SDS-PAGE indicated that cleavage was complete after 1 h and led to partial clearing of the solution, indicative of some droplets dispersing following cleavage (Figures S20 and S21). Examination of the remaining droplets by confocal microscopy indicated a marked maturation effect, with coarsening and roughening of their surfaces (Figures 4A and S22). TEM of the mature droplets confirmed the differences between these assemblies.

**Figure 4:**
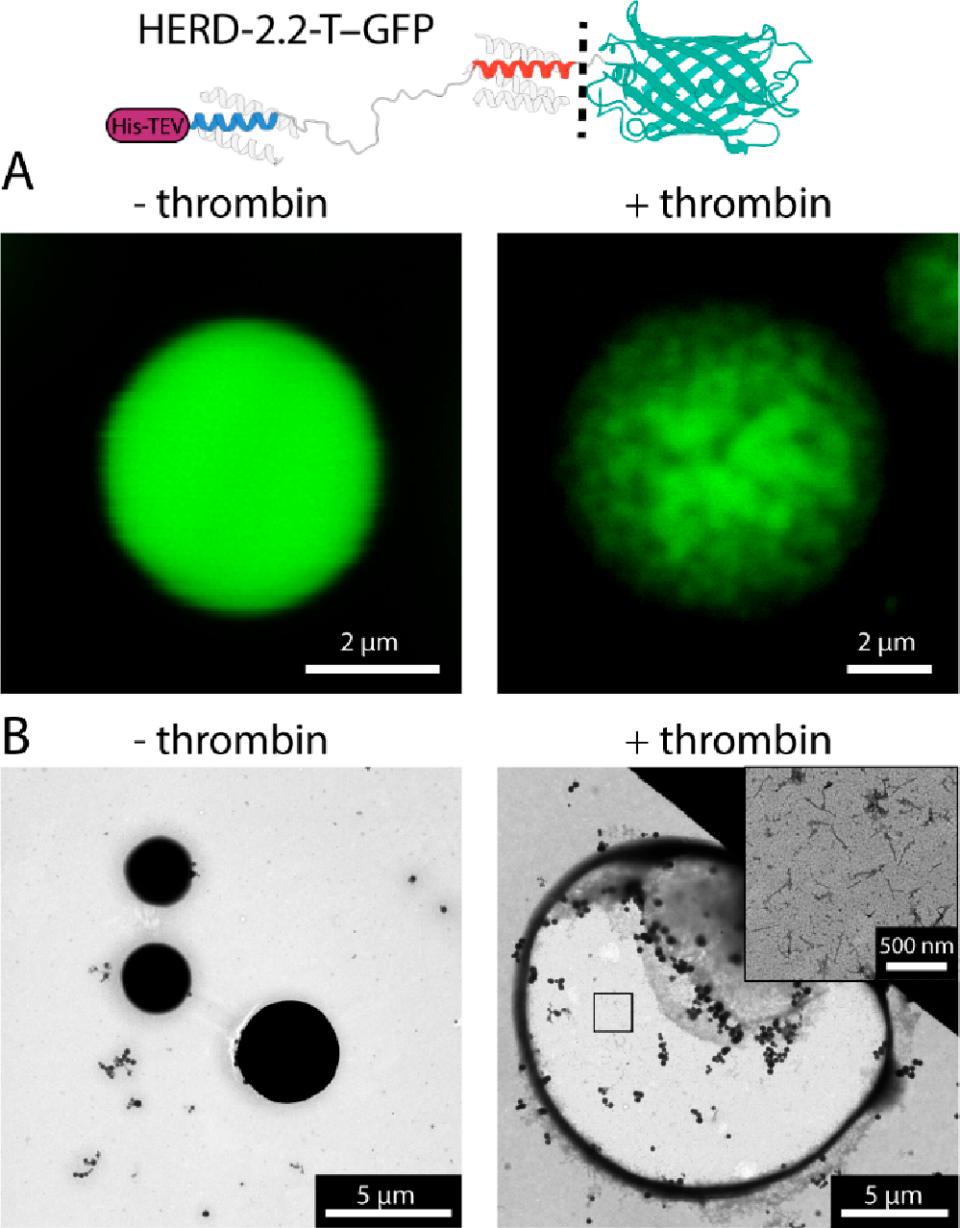
Maturation and fiber formation from HERD-2.2-T–GFP droplets. Confocal fluorescence microscopy (A) and TEM (B) of HERD-2.2-T-GFP with and without the protease. Conditions: 10% PEG 3350, 125 mM NaCl, 50 mM Tris pH 7.5, 0.675 mM HERD-2.2-T–GFP with (+ thrombin) or without (− thrombin) addition of 0.06 units of thrombin protease.

In the absence of protease, HERD-2.2-T–GFP formed highly dense droplets without evident internal structure (Figures 4B and S23). Following protease cleavage, however, the droplets formed hollowed-out macroscopic assemblies with a visible proteinaceous corona. Moreover, within these assemblies, rod-shaped fibers were observable, similar to those formed by HERD-2.2. Thus, the transition from demixed liquid to fiber can be triggered by proteolytic cleavage.

In summary, we have characterized a synthetic polypeptide, HERD-2.2, that drives the formation of both phase-separated droplets and macromolecular fibers, which assemble both *in vitro* and in cells. Previously, we have used LLPS of HERD-2.2–GFP to colocalize functional enzymes, conferring a boost to a two-enzyme pathway.^19^ Here, we demonstrate that HERD-2.2 fibers can also act as recruitment scaffolds in cells. We have identified that the *de novo* polypeptide can be switched between demixed droplets, and mature fibrous structures, and that switching is associated with a conformational change to β secondary structure. A switch from α to β secondary structure has been reported previously in other *de novo* designed peptides.^30^ The phase-separating protein TDP-43 also develops β structure during early droplet maturation events, eventually forming an amyloid-like state,^31^ and phase separation promotes β structure in Tau repeats.^32^ There are indications that a similar process may occur in FUS, which forms hardened β shells during droplet aging.^33^ This suggests that a similar process in the *de novo* HERD-2.2 may be recapitulating intrinsic properties of phase-separating proteins. All of TDP-43, Tau, and FUS form pathological aggregates linked to the neurodegenerative disorders Alzheimer’s, FTD, and ALS.^34−36^ The HERD-2.2 system could be of use in mimicking and following how droplet maturation occurs at the molecular level, providing insights into the nucleation and growth of pathogenic aggregates. Also, as demonstrated here, HERD-2.2 assembly in living cells makes them interesting for studying the effects of supramolecular polypeptide structures on cell biology.

## Supporting Information Available

Experimental materials and methods, protein sequences, additional microscopy images, CD data, and fiber diffraction patterns (PDF)
